# Malignant infarction of the middle cerebral artery in a porcine model. A pilot study

**DOI:** 10.1371/journal.pone.0172637

**Published:** 2017-02-24

**Authors:** Fuat Arikan, Tamara Martínez-Valverde, Ángela Sánchez-Guerrero, Mireia Campos, Marielle Esteves, Dario Gandara, Ramon Torné, Lidia Castro, Antoni Dalmau, Joan Tibau, Juan Sahuquillo

**Affiliations:** 1 Department of Neurosurgery, Vall d’Hebron University Hospital, Universitat Autònoma de Barcelona, Barcelona, Spain; 2 Neurotraumatology and Neurosurgery Research Unit (UNINN), Vall d’Hebron Research Institute (VHIR), Universitat Autònoma de Barcelona, Barcelona, Spain; 3 Experimental Surgery Unit, Vall d’Hebron Research Institute (VHIR), Universitat Autònoma de Barcelona, Barcelona, Spain; 4 IRTA, Animal Breeding and Genetics Program, Monells, Girona, Spain; "INSERM", FRANCE

## Abstract

**Background and purpose:**

Interspecies variability and poor clinical translation from rodent studies indicate that large gyrencephalic animal stroke models are urgently needed. We present a proof-of-principle study describing an alternative animal model of malignant infarction of the middle cerebral artery (MCA) in the common pig and illustrate some of its potential applications. We report on metabolic patterns, ionic profile, brain partial pressure of oxygen (PtiO_2_), expression of sulfonylurea receptor 1 (SUR1), and the transient receptor potential melastatin 4 (TRPM4).

**Methods:**

A 5-hour ischemic infarct of the MCA territory was performed in 5 2.5-to-3-month-old female hybrid pigs (Large White x Landrace) using a frontotemporal approach. The core and penumbra areas were intraoperatively monitored to determine the metabolic and ionic profiles. To determine the infarct volume, 2,3,5-triphenyltetrazolium chloride staining and immunohistochemistry analysis was performed to determine SUR1 and TRPM4 expression.

**Results:**

PtiO_2_ monitoring showed an abrupt reduction in values close to 0 mmHg after MCA occlusion in the core area. Hourly cerebral microdialysis showed that the infarcted tissue was characterized by reduced concentrations of glucose (0.03 mM) and pyruvate (0.003 mM) and increases in lactate levels (8.87mM), lactate-pyruvate ratio (4202), glycerol levels (588 μM), and potassium concentration (27.9 mmol/L). Immunohistochemical analysis showed increased expression of SUR1-TRPM4 channels.

**Conclusions:**

The aim of the present proof-of-principle study was to document the feasibility of a large animal model of malignant MCA infarction by performing transcranial occlusion of the MCA in the common pig, as an alternative to lisencephalic animals. This model may be useful for detailed studies of cerebral ischemia mechanisms and the development of neuroprotective strategies.

## Introduction

Stroke is the second most common cause of death and the third most common cause of disability-adjusted life years worldwide[1]. One-third of strokes occur in children and young and middle-aged adults[1], ischemic stroke (IS) being the most common subtype[2]. The rationale for aggressive therapy in IS is based on the fact that after acute ischemia, a variable amount of hypoperfused brain is at risk of permanent infarction (ischemic penumbra), but it may be potentially salvaged by early restoration of cerebral blood flow (CBF). The aim of translational research in IS is to improve neurological outcomes: it is the focus for basic science and clinical researchers, funding agencies, and the industry as a whole[3]. Despite remarkable advances in the understanding of the pathophysiology of ischemic lesions, however, ongoing efforts to identify novel molecular targets have not yet yielded new pharmacological therapies[4].

The term ‘malignant’ middle-cerebral artery (MCA) infarction was coined by Hacke et al. in 1996 to describe a form of IS that involved at least 50% of the MCA territory, followed an uniform clinical course, and resulted in transtentorial herniation and death in most patients despite optimal medical treatment[5]. To elucidate the pathophysiology of IS and develop neuroprotective therapies, animal models have been widely used. Despite limitations and ethical concerns, animal models are invaluable for investigating the pathogenesis of cerebral ischemia and evaluating the consequences of pharmacological intervention[6]. Since the early 1980s, the traditional animal model of IS has been occlusion of the MCA in the rat[7]. However, therapeutic strategies that appear efficacious in these experimental models have not been proven so when translated to patients. One explanation for this failure may be interspecies variability in cerebrovascular physiology, which may contribute to the divergent outcomes observed in rodent and human studies. The lysencephalic rodent brain is barely one-thousandth of the weight of the human brain and the proportions of grey and white matter also differ when comparing humans and rodents[8]. Humans, like other gyrencephalic species, have a considerably higher percentage of white matter (>60%) compared to lissencephalic species, such as rats or mice, which have only ~10%[8–10]. As a consequence of the recurrent failed translation to humans, the Stroke Therapy Academic Industry Roundtable (STAIR) recommended the use of large animal stroke models—e.g. pigs or nonhuman primates—before testing drugs or endovascular recanalization strategies in clinical trials[11].

Pigs have been described as excellent experimental animals for medical research because of the similarities between human and porcine biology. The pig brain is gyrencephalic and has a white-gray matter ratio similar to that of the human brain[10–12]. In a pivotal paper, Imai et al. presented a new, well-designed model of focal IS in the miniature pig that produced remarkable consistency in terms of infarct size, which was achieved by electrocoagulation of the 2 MCAs[13]. The aim of the present work is to present a proof-of-principle study describing a feasible large animal model of large hemispheric stroke in the common pig (*Sus scrofa domestica*) and to elucidate some of its potential advantages as an alternative to the few gyrencephalic models already available. Furthermore, this model will allow the use of all the modern neuromonitoring clinical tools that cannot be used easily in rodents.

In the present study we will show and discuss the results obtained using a porcine model. We characterized the volume of the infarction, the metabolic patterns of the ischemic tissue, the partial pressure of brain tissue oxygen (PtiO_2_), and the ionic profile of the extracellular space. We also explored at a molecular level the expression of sulfonylurea receptor 1 (SUR1) and transient receptor potential melastatin 4 (TRPM4), previously described in the brain of patients with multiple sclerosis [14], in subarachnoid hemorrhage[15], in cerebral infarcts[16], and in posttraumatic brain contusions[17]. TRPM4 is a calcium-activated nonselective cation channel that is expressed *de novo* after brain ischemia and injury. It is involved in the modulation of the brain immune response and the development of ischemic brain edema—and oncotic cell death—through the regulation of Ca2^+^ homeostasis, cationic fluxes, and membrane depolarization [14–18]. To our knowledge, this is the first study to explore these receptors in large IS-induced gyrencephalic mammals.

## Material and methods

### Experimental procedures and ethics statement

All procedures described in this study were approved by the animal experimentation ethics committee of the Vall d’Hebron Research Institute (protocol number 69/14) and were conducted in compliance with Spanish legislation and the directives of the European Union for animal research (2010/63/EU). All experiments were performed in 2.5-to-3-month-old female hybrid pigs (Large White x Landrace) weighing 30 to 40 Kg. Animals were sourced from A.M. Animalia Bianya S.L. animal center (Girona, Spain) and recorded in the registry of breeding centers, suppliers, and users of experimental animals with reference number G9900009. The transport company was authorized and certified to perform this service. Animals were previously acclimated to our facilities and housed in conventional pens for at least 1 week with free access to water and twice-daily feedings using a conventional diet. Animals underwent fasting for 12 hours before surgery but had free access to water with diluted sucrose. Only specimens with a satisfactory examination were included in the study. The pigs were kept under general anesthesia for the entire duration of the experiment and they did not experience any pain or distress.

### Housing conditions and diet

Environmental parameters were recorded and regulated daily. Temperature was maintained within a range of 19 to 21°C and humidity was 45 to 65%. Driven air was 100% external, pre-filtered, filtered with 95% efficiency, and processed with a renewal rate of 15 to 20 cycles per hour. The photoperiod was programmed for 12 hours of light and 12 hours of darkness, daylight hours being from 8:00 a.m. to 8:00 p.m. The animals had a minimum floor space of 0.5 m2. They were usually housed as a group of 4 to 6 animals with an area of 8 to 12 m2. Beds of wood shavings were provided, and for environmental and social enrichment we installed chains, balls, cylinders, hot plates of soil, an infrared plate in the ceiling, and alfalfa and sugar lumps as positive reinforcement.

The animals were monitored daily using the following criteria: appearance and body condition, observed behavior and habits, food and drink intake, and clinical follow-up after the procedure. Any anomalies or incidences were recorded. Observation of the animals was carried out by caregivers, technicians, research personnel, animal welfare advisers, and the veterinarians attending the animals. During the 24-hour period, a remote surveillance system involving a webcam was used to allow animals to be recorded and to study and assess their state, with the additional advantage of studying their behavior without the presence of people. In accordance with Spanish regulations (the Department of Agriculture, Food and Rural Affairs), all pigs went through a program of prevention and health surveillance and were vaccinated against Aujeszky disease.

### Anesthesia and analgesia

While still in the pen, animals were sedated with an intramuscular injection of 4 mg/kg of Tiletamine+Zolazepam (Zoletil 100^®^, Virbac SA, Esplugues del Llobregat, Barcelona, Spain) and 2 mg/kg of Xylacine (Xilagesic 20%^®^, Laboratorios Calier SA, Les Franqueses del Vallès, Barcelona, Spain). After loss of reflexes, the pigs were transported on a trolley. A venous catheter was placed in the auricular vein and an arterial catheter was placed in the auricular artery. Pre-oxygenation with 100% oxygen was applied using a facemask while the animals were aseptically washed. In the operating room, intravenous (IV) anesthesia was induced with Propofol (Propofol^®^, B Braun Medical SA, Melsungen AG, Germany) at 4 mg/kg. Endotracheal intubation was immediately performed and anesthesia maintained with 60% oxygen and 2% isoflurane (Isoflo, Abbott laboratories Ltd, Saint-Laurent, Québec, Canada) for the duration of the experiment. Ringer lactate (Ringer lactate, B Braun Medical SA) was continuously infused at 10 ml/kg/h and an IV constant-rate infusion of 6 **μ**g/kg/h of Fentanyl (Fentanest^®^, Kern Pharma SL, Terrasa, Barcelona, Spain) was also administered for analgesia. In order to prevent an increase in intracranial pressure (ICP), a single bolus of 10% mannitol (Fresenius Kabi España SA, Barcelona, Spain) was infused at 0.5 g/kg immediately before craniotomy.

### Physiological monitoring

Venous and arterial blood pressures were continuously monitored using arterial catheters placed in the cava vein and the femoral artery, respectively. Both catheterizations were performed through an ultrasound-guided technique. Anesthesia and ventilation parameters were controlled with an Aespire-7900^®^ device (GE Healthcare, General Electric Company, Fairfield, CT, USA), while heart rate, oxygen saturation, end-tidal CO_2_, and respiratory rate were monitored with an S/5^TM^ Compact anesthesia monitor Datex-Ohmeda^®^ (Datex-Ohmeda, Inc, Madison, WI, USA). A urinary catheter was also inserted. Core temperature was monitored with a rectal thermometer and controlled with a heating pad in order to maintain a stable core temperature of 38.5°C.

### Surgical approach and occlusion of the middle cerebral artery

**Fig 1:**
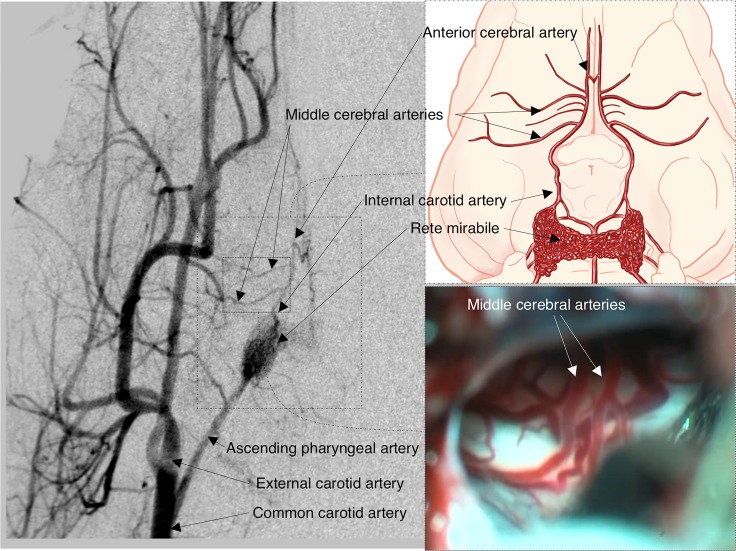
Basal vascular supply (1), angiographic study (2), and surgical view of the basal circulatory anastomosis (3) in the common pig. The most relevant arteries in the pig are shown in the angiographic study. Note that the animal presents a network of small bilaterally interconnected vessels called rete mirabile, the site from which the internal carotid artery originates intracranially. Rete mirabile are perfused on both sides by the ascending pharyngeal artery, which originates from the common carotid artery. In contrast to humans, it should be noted that in each hemisphere 2 middle cerebral arteries (MCAs) emerge from the internal carotid arteries, 1 coursing laterally and the other rostrally (the latter provides vascularization to the olfactory tract).

A frontotemporal approach was performed in the lateral position according to the surgical description by Imai et al.[13]. A curved skin incision was performed starting at the zygoma and extending to just above the right orbit. The frontotemporal bone and the orbital rim were exposed. Craniotomy was performed with a single burr hole made on the anterior aspect of the superior temporal line and then expanded using a Kerrison rongeur. The bone in the lateral part of the roof of the orbit was aggressively removed to facilitate access to the basal cisterns. The dura mater was opened with a semicircular flap. Two different brain regions were monitored intraoperatively by placing a polarographic Clark-type electrode (CC1.2 sensor, Integra Neurocare, Plainsboro, NJ, USA) and a CMA microdialysis probe (CMA-71, 8010320, M Dialysis AB, Solna, Sweden). The first area was labeled as the core area (CORE), with probes placed in the rostral sylvian gyrus of the frontal lobe corresponding to the vascular territory of the MCA in the common pig[13, 19]. These catheters recorded brain oxygenation and metabolism of the infarction area induced by the occlusion of the MCA. The second area, labeled the penumbra area (PENUMBRA), was monitored with 1 microdialysis probe placed in the ectosagittal rostral gyrus of the frontal lobe corresponding to a border area of the vascular territory between the MCA and the anterior cerebral artery (ACA)[13, 19]. Once the dura was opened, the operating microscope was positioned and a brain retractor was gently used to open the arachnoid and expose the basal cisterns, the optic nerve, the internal carotid artery, and the 2 MCAs. Ischemia was induced by clipping both MCAs (Fig 1), after which a control angiography was performed. Using a right femoral approach and a 5-French intravascular sheath, the carotid artery was catheterized with a 5-French intra-arterial catheter (Tempo 5, Cordis Corporation, Miami, FL, USA) to anatomically confirm the correct clipping of the 2 MCA branches. Euthanasia was scheduled at least 5 hours after the cerebral infarction was established: pigs received an IV dose of 2 g of thiobarbital (Thiopental, B Braun Medical SA).

### Brain tissue oxygen monitoring and microdialysis

Polarographic Clark-type electrodes (CC1.P1 sensor, Integra Neurocare) were connected to a tissue oxygen pressure monitor (Licox^®^ CMP system, Integra Neurocare), and the data obtained throughout the entire PtiO_2_ monitoring period were stored in a laptop and exported to a flat file for statistical analysis. The main objective of using these probes was to obtain real-time brain oxygenation levels and confirm that the surgical procedure was successful in establishing a reliable ischemic model with a severe drop in PtiO_2_. Cerebral MD probes were perfused with a sterile isotonic central nervous system fluid containing 147 mmol/L of NaCl, 1.2 mmol/L of CaCl_2_, 2.7 mmol/L of KCl, and 0.85 mmol/L of MgCl_2_ (P000151, M Dialysis AB, Stockholm, Sweden) at a fixed flow rate of 0.3 **μ**L/min using a microinfusion pump (CMA-402, M Dialysis AB). During the non-ischemic monitoring period (basal), microvials were changed every 30 minutes, while during ischemia microdialysate samples were collected every 60 minutes until death. Lactate ([Lac]_brain_), pyruvate ([Pyr]_brain_), glucose ([Glu]_brain_), and glycerol ([Gly]_brain_) were monitored using the point-of-care IscusFlex analyzer (M Dialysis AB). After hourly measurements were completed, microvials were placed on a rack designed to seal them and prevent evaporation (M Dialysis AB). All racks were stored at -20°C until ion analysis was carried out.

### Histological examination and infarct volume assessment

**Fig 2:**
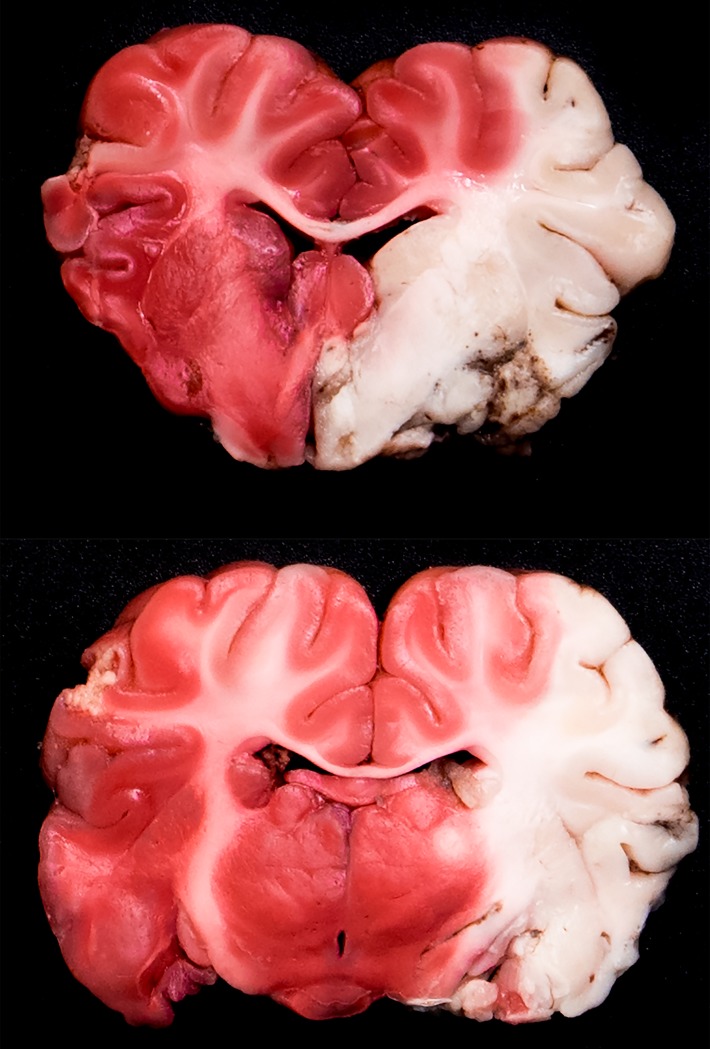
Two representative 5-mm brain coronal slices from animal #3 stained with 1% 2,3,5-Triphenyltetrazolium chloride (TTC) solution and showing the ischemic core at 7.5 h after left MCA occlusion. Note how with TTC staining the entire territory of the left MCA (i.e. the infarcted territory) remains a pale cream or white color, while the non-infarcted viable brain stains red or pink. In this particular case, the entire MCA territory was affected, including the 3 MCA sub-territories (deep, superficial anterior, and posterior). The deep territory of the MCA includes the caudate nucleus, the internal and the external capsules, the preoptic area, and the hypothalamus.

Immediately after death, brains were carefully extracted and placed on ice for 15 minutes. Microdialysis and PtiO_2_ probes were not removed in order to identify the exact insertion site. Five-mm coronal slices were obtained and stained with 2,3,5-triphenyltetrazolium chloride (TTC; 93140; Sigma-Aldrich, Inc., St. Louis, MO, USA) to determine the infarct volume. Each brain slice was placed in a 60-mm dish, covered with 1% TTC solution (dissolved in 0.9% saline), and incubated at 37°C under dark conditions for 30 minutes. Using TTC staining, viable gray matter was stained red or pink and infarcted tissue remained a pale cream or white color (Fig 2). Next, both sides of each section were rinsed twice in 0.9% saline solution and fixed with 4% formol for 7 days. The placement of the catheters was identified post-mortem and classified using 3 possible categories: infarct core, ischemic penumbra, and healthy brain. All brain slices were photographed (Nikon D750, Nikkor 50mm Lens, Nikon and Essilor International Joint Research Center Co., Ltd., Kanagawa, Japan). For infarct volume assessment, TTC images from both sides of the brain slices were digitalized by using a flatbed scanner (HP Scanjet G4010, Hewlett Packard Enterprise, Sant Cugat del Valles, Barcelona, Spain). The final infarct volume of all animals was quantified by 1 of the investigators (TMV) using ImageJ software (Wayne Rasband, National Institutes of Health, USA). We calculated the volume of the infarct by adding the infarct volumes of each cut, which were obtained by multiplying the mean of the infarct area (the area that remains pale cream or white after TTC staining) of the anterior and posterior surface of each sample by the thickness of each slice. The total volume of the supratentorial brain was also obtained using the same software to sum up the volume of each slice (also obtained by multiplying the thickness of each slice by the mean of the anterior and posterior areas). The infarct volume was expressed in cm^3^ and as a percentage of the total volume of the brain in each animal.

### Immunohistochemistry

For immunohistochemistry analysis, samples from the different areas (core, penumbra, and healthy contralateral brain) were obtained. All samples were dehydrated with different alcohol solutions (70°, 90°, and 100°). Brain tissue samples were cryoprotected using 30% sucrose and embedded in a Tissue-Tek optimal cutting temperature compound (4583; Sakura Finetek Europe B.V, Alphen aan den Rijn, The Netherlands). From these blocks, 10-μm sections were obtained using a cryostat (Leica CM3050 S; Leica Biosystems, Heidelberg, Germany), mounted on glass slides, and stored at -20°C until analysis was carried out. For immunohistochemistry analysis, sections were incubated in a blocking solution containing 2% donkey serum (D9663; Sigma-Aldrich) and 0.2% Triton-X (T8787; Sigma-Aldrich) in 0.1 M phosphate-buffered saline for 1 h. Next, cryosections were incubated for 1 hour at room temperature and then for 48 hours at 4°C with the primary antibodies goat anti-SUR1 1:100 (Santa Cruz Biotechnology, Santa Cruz, CA, USA) and chicken anti-TRPM4 1:500 (custom anti-TRPM4 antibodies described by Woo et al.[2]). Fluorescent-labeled, species-appropriate secondary antibodies (Invitrogen™, Eugene, OR, USA) were used for visualization. Omission of primary antibodies served as a negative control. Sections were cover-slipped with polar mounting medium containing antifade reagent and the nuclear dye 4,6-diamino-2-phenylindole (P36935; Invitrogen). Fluorescent signals were visualized using an epifluorescence microscope (BX61 Olympus; Olympus Corporation, Tokyo, Japan).

### Quantitative immunohistochemical analysis in neurons and vessels

To calculate SUR1/TRPM4-positive neurons and endothelial cells, between 4 and 8 randomly captured 440×330-μm2 images from the cortex (NEURONS) and the white and grey matter (VESSELS) were taken with an epifluorescence BX61 Olympus microscope. The primary antibodies used were mouse anti-NeuN 1:100 (MAB377, Millipore Corporation, Billerica, MA, USA) for neurons and CD31: mouse anti-CD31 1:100 (M082329; Dako, Carpinteria, CA, USA) for vessels. Next, all images were quantified using the plugin Cell Counter (Kurt De Vos; http://rsb.info.nih.gov/ij/plugins/cell-counter.html) from the Image J 1.47v program (Wayne Rasband, National Institutes of Health, Bethesda, MD). Using the total number of neurons and vessels (NeuN and CD31-positive cells, respectively), the percentage of SUR1/TRPM4-positive cells was calculated.

### Semi-quantitative immunohistochemical analysis in astrocytes

To evaluate SUR1 and TRPM4 expression in the astrocytes, we used an anti-GFAP 1:3000 mouse antibody (C9205, Sigma-Aldrich). Next, the whole section was quantified by a single observer using a semi-quantitative scale to count the following: 1) GFAP-positive cells (0: absent, 1: scant, 2: moderate, and 3: numerous) and 2) SUR1-positive cells and TRPM4-positive cells of each type (0: none; 1: in a few cells; 2: in many cells, and 3: in almost all or all cells).

### Ionic profile of the extracellular space

After hourly microdialysis measurements were completed, the microvials were placed in a rack designed to seal them and prevent evaporation (MDialysis AB). All racks were stored at -20°C until analysis could be performed. Prior to analysis, the microvials were defrosted on ice and the ionic profile was determined using an ICP-MS analyzer (Agilent 7500ce, Agilent Technologies, Santa Clara, CA, USA) with collision cell technology using He as inert gas at 5 mL/min. All concentrations obtained were corrected using a previously-defined linear model described previously[20].

### Statistical analysis

Data were analyzed and summarized using the SPSS program for Mac (Version 20, SPSS, Inc., New York, USA). Because most variables followed a non-normal distribution, data were summarized using the median, minimum, and maximum. Immunohistochemical findings of SUR1 and TRPM4 were compared using nonparametric tests and statistical significance was defined as *p*≤0.05. Graphics were created using R v3.2.0 (R Foundation for Statistical Computing, Vienna, Austria; http://www.R-project.org) and the integrated development environment R Studio v0.99.903 (RStudio, Inc., Boston, MA, USA; http://www.rstudio.com).

## Results

### Ischemic period and infarct volume

A total of 5 animals were included in the study. Two of the 5 animals (#1 and #4) died early after ischemic induction and did not complete the study protocol. Both animals presented severe hypotension 30 min after MCA occlusion that could not be reverted with fluidotherapy and vasoactive drugs, resulting in cardiac arrest before the experiment could be completed. Despite active resuscitation, animal #1 died 4.5 hours after MCA occlusion and in animal #4 death occurred 4 hours after MCA clipping. The median ischemic time achieved in our study was 6.5 hours (min: 4, max: 7.5 h). The median infarct volume was 12.3 cm^3^ (min: 6.6, max 15.8 cm^3^), representing on average 18.4% of the total brain volume (min: 9.5%, max: 25.1%). Table 1 summarizes data on the ischemic period and the infarct volume for all animals. Animals with a larger infarcted area (14.2 cm^3^ in animal #3 and 15.8 cm^3^ in animal #5) had a longer clipping time (7.5 h and 7 h respectively). Animal #4, which had a shorter ischemic period (4 h), had the lowest volume of infarcted tissue (6.6 cm^3^).

**Table 1:** Ischemic period and infarct volume in pig specimens.

Animal	Ischemic period (h)	Infarct volume (cm^3^)	Infarct volume (%)
1	4.5	12.3	18.4
2	6.5	9.1	13.8
3	7.5	14.2	23.6
4	4	6.6	9.5
5	7	15.8	25.1

TTC images from both sides of the brain slices were digitalized and the final infarct volume was quantified by adding the volume of each slice, which was obtained by multiplying the thickness by the mean area of the anterior and posterior areas of each slice.

### PtiO_2_ monitoring

PtiO_2_ values are summarized in Table 2. With the exception of animal #5, PtiO_2_ monitoring was very reliable in confirming the establishment of the infarction. PtiO_2_ levels dropped immediately after clipping the arteries, reaching minimum values approximately 60 min after clip placement. In a sole animal (#5), no changes in PtiO_2_ were observed after MCA occlusion, despite confirmation by cerebral angiography of the correct clipping of the 2 branches. In this animal, post-mortem study revealed that the PtiO_2_ probe had been placed outside the area of infarction, while an angiographic control confirmed the occlusion of both MC arteries. PtiO_2_ data for this animal was excluded from statistical analysis, but its metabolic data was used.

**Table 2:** Metabolite values and PtiO_2_ measurements in the entire animal group during basal and ischemic periods in CORE and PENUMBRA.

Condition	TIME	PtiO_2_ (mmHg)	MICRODIALYSIS CORE (n = 5)	MICRODIALYSIS PENUMBRA (n = 3)	MICRODIALYSIS PENUMBRA (n = 3)	MICRODIALYSIS PENUMBRA (n = 3)	MICRODIALYSIS PENUMBRA (n = 3)	MICRODIALYSIS PENUMBRA (n = 3)	MICRODIALYSIS PENUMBRA (n = 3)	MICRODIALYSIS PENUMBRA (n = 3)	MICRODIALYSIS PENUMBRA (n = 3)	MICRODIALYSIS PENUMBRA (n = 3)
**BASAL OR ISCHEMIA**	(h)	CORE (n = 4)	Glucose (mM)	Lactate (mM)	Pyruvate (mM)	LPR	Glycerol (μM)	Glucose (mM)	Lactate (mM)	Pyruvate (mM)	LPR	Glycerol (μM)
BASAL	0	27.5 (24.4–39.2)	1.59 (0.92–2.25)	1.8 (1.2–2.5)	0.101 (0.072–0.131)	17.4 (16.1–18.8)	44.2 (23.0–65.5)	2.72	2.40	0.243	9.88	15.1
ISCHEMIA	1	0.45 (0.0–3.2)	0.70 (0.14–2.08)	8.18 (1.56–13.4)	0.074 (0.016–0.142)	64.9 (19.5–853)	77.6 (37.5–217)	1.22 (0.55–2.19)	4.54 (4.40–7.32)	0.245 (0.019–0.321)	29.9 (13.72–244.5)	148 (119–244)
ISCHEMIA	2	2.8 (1.0–4.6)	0.09 (0.00–1.88)	8.25 (7.25–9.85)	0.015 (0.000–0.050)	3241 (27.2-N/A)	319 (116–544)	1.40 (1.13–1.84)	4.06 (2.62–4.97)	0.179 (0.096–0.379)	27.2 (10.7–27.7)	103 (98.5–507)
ISCHEMIA	3	2.2 (1.0–3.1)	0.18 (0.00–2.59)	7.29 (6.85–9.43)	0.011 (0.000–0.047)	3225 (187-N/A)	462 (187–734)	1.21 (1.01–1.64)	2.94 (2.58–3.40)	0.261 (0.095–0.292)	11.7 (9.89–31.2)	111 (90.2–660)
ISCHEMIA	4	0.0 (0.0–4.7)	0.15 (0.00–2.46)	6.68 (5.84–10.7)	0.008 (0.000–0.034)	6282 (315-N/A)	545 (306–794)	1.13 (0.29–1.15)	3.06 (1.91–4.85)	0.212 (0.062–0.251)	22.9 (7.6–49.5)	115 (75.8–882)
ISCHEMIA	5	2.0 (0.0–3.4)	0.03 (0.00–2.02)	8.87 (5.16–11.2)	0.003 (0.000–0.032)	4202.0 (346-N/A)	588 (311–790)	1.13 (0.05–1.24)	2.62 (1.98–8.13)	0.251 (0.023–0.298)	27.3 (7.87–116)	141 (73.3–751)

Ischemia values are expressed as median (minimum-maximum). PtiO_2_ data of animal #5 was excluded from statistical analysis after confirming in the post-mortem study that the PtiO_2_ probe had been placed outside the area of infarction. For the sake of clarity, values under detection were considered to be 0, although the lower detection limits were 0.1 mmol/L for glucose and 0.01mmol/L for pyruvate. When pyruvate was under this limit, an L/P index was not calculated (N/A).

Basal levels in the penumbra area were only available in the animal #5, and therefore minimum and maximum values in the penumbra area were not available in the basal determination.

### Brain microdialysis monitoring

Values for the different metabolites measured at baseline and after ischemia in the core and penumbra are summarized in Table 2. A cerebral MD catheter was implanted in the ischemic core in all the animals and in the ischemic penumbra in the final 3 animals.

#### Changes in the core

**Fig 3:**
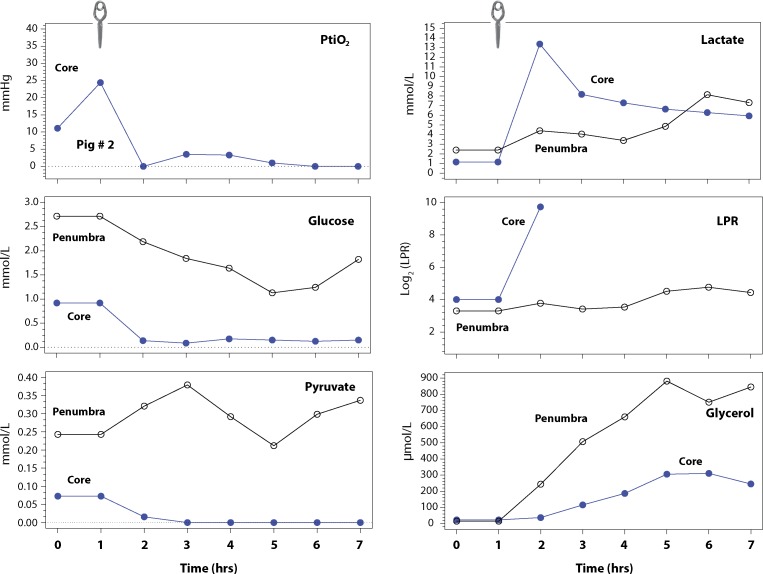
The representative pattern of microdialysis values in the ischemic core and in the penumbra of animal #5. We used PtiO_2_ data from animal #2 to represent the PtiO_2_ drop after clipping both MCAs because the probe had been misplaced outside the core in animal #5 (image not shown) and because baseline data was missing in some of the remaining animals. The increase in PtiO_2_ in the first 2 hours may be explained by the running time of PtiO_2_ probes. The clip illustrated at the top of the diagram shows the time in which clipping of both MCAs was carried out. PtiO_2_ data in animal #2 was consistent with the typical PtiO_2_ profile observed in all but 1 animal (#5). In the ischemic core, a rapid decrease in [Glu]_brain_ was observed after occlusion, followed by a significant drop in [Pyr]_brain_ and a significant increase in [Lac]_brain_ and in the lactate/pyruvate ratio (LPR). LPR values 2 hours after clipping could not be calculated because [Pyr]_brain_ levels were undetectable and therefore LPR rose to infinite values. A significant increase in [Pyr]_brain_ was also observed in the core, reaching a plateau at 5 h post-ischemia. In the penumbra area, [Lac]_brain_ and the LPR values also increased, but they were not as pronounced as in the core. [Pyr]_brain_ levels were unstable in the penumbra and at 4 to 7 h after clipping followed the same pattern as [Glu]_brain_. Glycerol also increased in the penumbra, reaching levels well above those observed in the samples taken from the core.

A significant decrease in [Glu]_brain_ in the core was observed in all animals soon after occlusion, with the exception of animal #4. Post-mortem analysis of the brain of this animal showed that the cerebral MD probe had been placed in an infarcted area, but there had been significant collateral circulation around the necrotic tissue that might explain the observed readings in [Glu]_brain_. In all animals, the drop in [Glu]_brain_ was followed by a significant drop in [Pyr]_brain_ to undetectable levels and a significant increase in [Lac]_brain_, with a consequent increase in the lactate/pyruvate ratio (LPR) (Fig 3 and Table 2). A typical example of the metabolic changes observed in both the core and the penumbra in 1 animal (#5) is shown in Fig 3. A significant increase in [Gly]_brain_ was observed in the core of all animals and reached a maximum value of 794 μmol/L in 1 animal.

#### Changes in the penumbra

Heterogeneous changes were found in all metabolites for the penumbra, showing a variable degree of metabolic stress (Table 2) or even progression to non-viable tissue at the end of the experiment in 2 animals (#3 and #4). The small number of animals in this pilot study precludes a statistical analysis of the metabolic profile of the penumbra, however it may be noted that a significant reduction in [Glu]_brain_ (~50%) with respect to the baseline values was observed in most animals. In 2 animals (#3 and #4) the decrease was more marked at the end of the experiment, indicating recruitment of the penumbra into the core. [Lac]_brain_ increased in the penumbra, but it was less marked than in the core (Fig 3 and Table 2). The same trend was observed with LPR. [Gly]_brain_ also increased in all animals but did not reach the levels observed in the core, with the exception of animal #5.

### Ionic profile of the extracellular space

After MCA clipping, ionic data were obtained hourly for the entire period in both brain regions. Due to the minimum required dialysate for the ionic analysis, it was not possible to obtain the medians, minimums, and maximums of the 3 ions during the baseline period. During the ischemic period the ionic profile in the core was compiled from 24 valid determinations, and in the penumbra area the ionic profile was made from 19 valid determinations. The ionic profile was significantly different for each monitored brain area. The ischemic core was characterized by an increase in median K^+^ to 27.9 mmol/L (min: 4.0, max: 49.4), while penumbra presented increased Na^+^ levels (median 164.0 mmol/L; min: 130.7, max: 208.0). Table 3 summarizes the ionic data of the extracellular space.

**Table 3:** Ionic data of the extracellular space in both monitored brain areas.

Monitored area	[Na^+^] mmol/L	[K^+^] mmol/L	[Cl^-^] mmol/L
**Ischemic core**	154.1 (135.3–199.8)	27.9 (4.0–49.4)	157.0 (132.3–187.0)
**Ischemic penumbra**	164.0 (130.7–208.0)	7.5 (3.7–44.0)	157.7 (147.2–208.0)

Values are summarized as median (min-max). Due to the minimum required volume for ion analysis, the median, minimum, and maximum values of the 3 ions in the core were made from 24 valid determinations, and in the penumbra area they were made from 19 valid determinations.

### SUR1-TRMP4 expression

**Fig 4:**
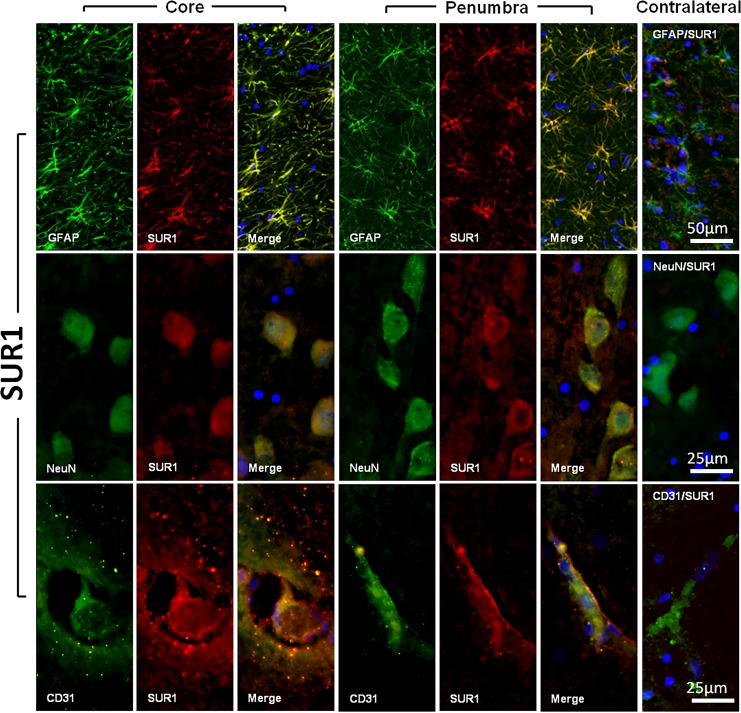
SUR1 expression in astrocytes, neurons, and capillary endothelial cells. The figure shows fluorescent double labeling for GFAP (panel A), NeuN (panel B), CD31 (panel C) and SUR1 in the core and penumbra regions. The most lateral column on the right shows the merged images of the controls (i.e. contralateral healthy tissue). Original magnification = 20×(A) and 40× (B, C). Nuclei were counterstained with DAPI. All tissue sections were obtained from animal #5 7 hours after ischemia onset.

All sections were examined by a single observer (LC), who carried out a quantitative or semi-quantitative analysis, depending on the cell type, for SUR1 and TRPM4 expression (Fig 4). In neurons, immunofluorescence showed that the expression of both the regulatory subunit (SUR1) and the pore forming subunit (TRPM4) were significantly increased in the neurons in the penumbra area and in the ischemic core when compared with the contralateral healthy hemisphere (Kruskal-Wallis, *p* = 0.01 in both cases). A summary of the semi-quantitative findings for SUR1-positivity and TRMP4-positivity are shown in Table 4. A mild overexpression of SUR1 and TRPM4 was detected in the GFAP-positive cells in the normal brain tissue samples. Both the penumbra and core samples presented strong SUR1 overexpression, with moderate expression of the TRPM4 channel (Fig 4). For endothelial cells (CD31+ cells), we did not find any significant difference in SUR1 expression when comparing the core, the penumbra, and the contralateral healthy tissue. However, we did find a significant difference in TRPM4 expression when comparing the 3 different regions in all cells (Kruskal-Wallis, *p* = 0.02) (Fig 4 and Table 4).

**Table 4:** Expression of SUR1 and TRPM4 in neurons and vessels. Results are shown as median (min–max) of the percentage of SUR1/TRPM4-positive neurons and vessels versus the total number in these 2 cell types.

Cell type	SUR1 expression (%) Contralateral	SUR1 expression (%) Penumbra	SUR1 expression (%) Core	TRPM4 expression (%) Contralateral	TRPM4 expression (%) Penumbra	TRPM4 expression (%) Core
Neurons	3.63 (0.00–22.3)	52.0 (18.6–62.6)	79.3 (66.9–86.8)	4.72 (0.00–19.9)	32.9 (22.4–37.5)	58.5 (40.1–64.5)
Vessels	40.0 (4.55–55.7)	65.3 (51.2–83.9)	72.8 (32.2–88.7)	61.3 (43.7–79.6)	77.8 (75.6–79.0)	87.2 (83.3–91.1)

## Discussion

Most treatments used to manage patients with IS (e.g. intravenous rtPA, intra-arterial treatments, and mechanical thrombectomy) seek to obtain rapid recanalization of the occluded artery and reperfuse the ischemic brain to reduce the final amount of necrotic brain[3, 21]. However, no effective neuroprotective strategies have been found to reduce either brain edema or the amount of ischemic brain that is recruited to the core once the attempt to reopen the occluded artery has failed. Sudden deprivation of oxygen and glucose to the brain elicits a series of pathological cascades that contribute to the affected brain tissue’s progression to necrosis. Excitotoxicity, metabolic derangements, tissue acidosis, accumulation of intracellular calcium cations, neuroinflammation, excessive production of free radicals, disrupted BBB and brain edema, apoptosis, and the overexpression of channels involved in regulating sodium and potassium all play critical roles in ischemic damage and in the deterioration of the penumbra[22]. Many drugs and agents that have been shown to reduce the infarct size in animal models—mostly in rodents—have failed dramatically in the clinical arena[4, 22]. This is particularly relevant for patients with malignant IS for whom space-occupying brain edema is the most important cause of death and disability[23] and the only treatment option is to conduct decompressive craniotomy to reduce the dismal mortality they nonetheless present with maximal medical treatment[23]. Among the many reasons suggested for the clinical trial failures, 1 very important factor is the lack of an animal model to reproduce the complex cascades observed in humans after IS[22]. To date, most experimental animal models use animals with lissencephalic brains, such as the mouse or the rat. The proportions of grey and white matter in rodents differ from that of humans. Gyrencephalic species have a higher percentage of white matter than lissencephalic species[8, 9], an anatomical difference that may lead to different mechanisms of cellular injury and recovery[24]. Recent studies have shown that in patients with stroke, the degree of affected motor function is more closely related to white matter integrity than to the BOLD response of cortical motor areas[25]. In addition, aquaporin-4 (AQP-4)—a main player in ischemic brain edema—is exclusively expressed by astrocytes. Stokum et al. have shown in a rodent experimental model of ischemia that subcortical white matter is much more susceptible to post-ischemic tissue swelling than cortical grey matter, and that cortical astrocytes exhibit unchanged expression in AQP-4, while white matter astrocytes exhibit a significant increase in AQP-4 expression after induced ischemia[26]. Those authors confirmed their findings in humans and suggested that white matter may play an underestimated active role in the formation of cerebral edema following ischemia[26].

There is a wide consensus that developing new therapeutic strategies in animal models is crucial despite the ethical concerns that animal experimentation raises. As some authors emphasize, ethical issues and animal welfare constitute an important limitation that should be clearly weighed against their scientific potential, medical benefit, and the availability of appropriate alternative approaches[27, 28]. For animal experiments on cerebral ischemia, Kuroiba and Okeda described the criteria that should be considered when selecting an appropriate species to ensure the findings have clinical relevance[8]. The animal whose brains are closest to human brains are non-human primates. However, modelling brain disorders in primates is very expensive, availability is limited, and relevant ethical considerations remain unresolved[28].

### The pig: A neglected animal model

In the rat, unilateral common carotid artery occlusion and intraluminal thread occlusion of the internal carotid artery are classic procedures for inducing focal brain ischemia without intracranial manipulation[8]. Transitioning IS modelling from rodents to large mammals with gyrencephalic brains that possess a white-gray matter ratio that is closer to the human brain is crucial for new preclinical models. Pigs are an alternative to primates as a non-rodent species because of their anatomical and physiological similarities to humans. The pig brain is only 7.5 times smaller than the human brain and is composed of >60% white matter[12, 22]. An added value of using pigs as experimental animals is that they are widely available due to commercial production and less constrained by ethical and economic considerations. Pigs have been widely used in toxicology and experimental surgery, but are only occasionally used in neuroscientific research, something that has progressively increased only in the past decade[12]. As Lind et al. report, most agricultural pigs are derived from the Eurasian wild boar (*Sus scrofa*) but there are a large number of breeds with significant anatomical differences[12]. In addition, pig-producing countries do not generally supply pure breeds, but rather crossbreeds of various recognized breeds (e.g. Landrace, Yorkshire, Hampshire, and Duroc)[29].

In modelling brain ischemia, researchers should be familiar with the important anatomic, histopathologic, and clinicopathologic features of the pig brain and neurovascular supply in order to reproduce a valid model of focal ischemia. Pigs, like other experimental large animals, have the disadvantage of having a prominent external carotid circulation from which a rete mirabilis is formed and from which the internal carotid artery originates (Fig 1)[13, 30]. This collateral circulation, described angiographically by Burbridge et al., has caused some authors to rule out swine for brain ischemic models. Rete mirabilis restricts the occlusion of the carotid artery and their branches by intravascular methods and the use of microcatheters[30]. However, this anatomical variant is also present in other mammals, such as cats, goats, dogs, and sheep[31].

The second important difference between pigs and humans is that the posterior communicating artery in pigs is comparable in size to the ICA. As a result, the connection between the anterior and posterior circulation systems is very well developed when compared with humans. An additional important difference is that in each hemisphere 2 MCAs originate from the ICA, 1 coursing laterally and another rostrally over the olfactory tract[13].

### Considerations when modelling focal brain ischemia in the pig

The same limitations raised by our group concerning the use of models in rodents led a team at the Aarhus University of Denmark to propose for the first time animal models of infarction using common pigs with transorbital occlusion of the MCA[32]. That group made important contributions to the understanding of the cerebrometabolic changes that occur after irreversible occlusion in pigs. However, in our opinion, that animal model was not introduced in more centers due to the complexity of surgical access, which is less common in neurosurgery. Another factor could have been that infarct volumes obtained in this animal model showed a greater variability, probably due to the non-complete occlusion of the entire territory of the MCA; the surgical corridor only gives access to 1 of the 2 branches of the MCA present in the pig[32–36].

The main anatomical consideration in the model of permanent arterial occlusion we present is that MCA occlusion involves an open surgical frontotemporal approach and the clipping of both MCAs. After a learning curve, the total surgical procedure took approximately 60 minutes. We believe this model has many advantages over the classic rodent model. We found that our model gives a satisfactory representation of malignant IS with a dense, reproducible infarction that causes early death if untreated. Moreover, the percentage of infarcted volumes we obtained from animal modeling appears to be consistent and is comparable to the volumes obtained in the classical models of malignant infarction in rats[37–40]. In addition, this model allows the use of conventional angiography to confirm optimal arterial occlusion. Like the minipig model presented by Imai et al., our model is a feasible, large gyrencephalic model of focal IS that may be more useful than alternative models of focal cerebral ischemia in medium gyrencephalic animals, such as dogs, cats, or even subhuman primates. The use of vascular clips instead of coagulation of both MCAs allows for the design of experimental models in which the clips can be removed at different times after ischemia, modelling temporary ischemia and allowing for the study of reperfusion phenomena. In addition, this model allows for the use of PtiO_2_ and cerebral MD probes to study the metabolic disorders induced by ischemia, as well as the ionic disturbances of the ischemic brain and its potential reversibility through use of various therapeutic strategies. The main disadvantages of using large animals are handling difficulties within experimental facilities and the increased workload for the research group resulting from multi-hour experiments. When considering which swine species to use when establishing an animal model, domestic pigs have several advantages over minipigs, including lower cost and greater availability.

### Metabolic and ionic profile in ischemia

Continuous PtiO_2_ monitoring is a reliable surrogate measure of rCBF and can detect ischemic and non-ischemic causes of brain hypoxia, such as low-extractivity hypoxia, shunt hypoxia, or dysperfusion hypoxia[41–43]. In all cases in which the probe was correctly inserted, PtiO_2_ levels dropped immediately and were thus a reliable indicator of the complete occlusion of both MCAs. After a careful anatomical examination of the brain in the only animal in which PtiO_2_ values did not change, the probe’s tip was found to be located outside the ischemic lesion. To our knowledge, this is the first report of the ionic profile monitored hourly together with energy metabolism in a brain IS, with results that are consistent with the patterns found in the human brain[44]. Malignant strokes cause massive ionic fluxes, with consequent osmotic water movement across cells and cerebral edema formation. Changes in ionic concentrations induce water accumulation in the intracellular and extracellular space and cause the injured brain tissue to swell, resulting in neurological worsening[45]. These ionic disorders are directly related to the overexpression of different ion channels that can be constitutive or newly synthesized, such as the SUR1-regulated channel TRPM4[16, 45]. TRPM4 belongs to a large family of proteins that share certain structural similarities. Most members of the TRP family are permeable to divalent cations. However, TRPM4 is impermeable to Ca2^+^ because it exclusively and non-selectively transports monovalent cations. In situations of ischemia there is an increase in the transcription of SUR1 that is accompanied by overexpression of TRPM4. TRPM4 channels are activated either by an increase in cytosolic Ca2^+^ or by a decrease in the ATP/ADP ratio in the cytosolic space. SUR1-TRPM4 is not constitutively present in cells of the central nervous system, but its transcription increases in neurons, the capillary endothelium, and astrocytes several hours after the onset of cerebral ischemia. In the absence of ATP this channel is activated, favoring edema and oncotic cell death due to the massive entry of ions[16, 18, 46].

The use of brain MD in large animal models offers a unique opportunity to observe the dynamic ionic changes in the brain over time and opens up a new path to explore the ionic profile during edema formation and its changes after different therapies.

From a metabolic perspective, our model allowed us to reproduce the changes expected in permanent focal ischemia and detect the metabolic deterioration of the penumbra (Fig 3). Furthermore, we found that [Gly]_brain_ increased with time and can be used in experimental models as a biomarker for the progression of the damaged brain. Glycerol is considered a biomarker of brain tissue damage and its concentrations rise during cellular energy failure and cell damage[47, 48]. However, it is presently unclear whether increased [Gly]_brain_ is associated with the destruction of the cell membrane and cell death, or whether it is a marker of cell "suffering" with the possibility of reversal. To clarify this distinction, further studies using the same experimental model are needed.

### Limitations of the study

The main limitation of our study was the small sample size inherent to most animal-based experimental studies. Both cost and ethical issues (ensuring animal welfare) constitute a limitation and the number of animals must be reduced to the minimum required to obtain answers to predefined scientific questions. The aim of this article is to present a proof-of-principle study, using a small sample of subjects as per Directive 2010/63/EU of the European Commission for the protection of animals used for scientific purposes. Due to its small sample size, our study did not try to look for statistically significant results, but rather described the feasibility of a large hemispheric stroke in the common pig and elucidated some of its potential advantages as an alternative to rodents. Therefore, we cannot rule out that increasing the sample size could introduce more variability in the final infarction size.

A second limitation is that our study was not designed to reproduce a reperfused focal infarction, and therefore we cannot present data on the lesions found after reperfusion or report on the time needed to obtain a complete infarction versus a reversible focal lesion. Our main goal was to determine the feasibility of obtaining a reproducible malignant infarction, and transient ischemia was not an endpoint in our model. Another drawback is that our model was designed to study only the early phenomena occurring at the very first stages of complete arterial occlusion, and therefore we cannot present data on the survival of animals, the degree of brain swelling—which in humans with malignant stroke develops many hours after the ischemic insult—or the long-term neurological sequelae that have been extensively studied in rodents that survive an IS. Finally, we must also highlight as a possible limitation of our study the need for anesthetic agents with possible neuroprotective effects, including propofol, zolazepam, and mannitol. However, we emphasize the difficulty in limiting their use in big-animal models since they are needed to facilitate microsurgical access of the cisterns for the clipping of both MCA branches, thus minimizing injuries secondary to the cerebral retraction.

## Conclusions

We report on the development of a porcine model of malignant IS involving craniotomy and the clipping of both MCAs as a feasible model that allows for the study of both ischemia-induced metabolic disorders and disturbances in the ionic profile. We believe that this model provides an excellent opportunity to better understand the mechanisms of cerebral ischemia in a human-like gyrencephalic brain and the neglected importance of white matter in post-ischemic brain edema, aiding in the development of novel therapeutics that can potentially be translated to patients. In addition, this model may help in elucidating the mechanisms that lead to the recruitment of the penumbra to the infarction core and the pathophysiology of ischemic brain edema. Furthermore, it will allow researchers to test new therapeutic strategies that, alone or in combination, may target some of the many molecular cascades (specifically, the SUR1-regulated TRPM4 channel) that may be useful in reducing edema-induced brain swelling and therefore improving the functional outcome of patients with malignant IS. Our animal model allows for the study of the temporal profile of TRPM4 overexpression in the ischemic brain, as well as the other ionic and water channels involved in focal brain ischemia. More importantly, it may open up a clear line of research on the effects of SUR1-antagonists as neuroprotective drugs in gyrencephalic animals, the brains of which are very similar to those of humans.

## Abbreviations

PtiO_2_: Partial pressure of brain tissue oxygen
SUR1: Sulfonylurea receptor 1
TRPM4: Transient receptor potential melastatin 4
MCA: Middle-cerebral artery
IS: Ischemic stroke
CBF: Cerebral blood flow
STAIR: Stroke Therapy Academic Industry Roundtable
MD: Cerebral microdialysis
IV: Intravenously
ICP: Intracranial pressure
ACA: Anterior cerebral artery
[Lac]_brain_: Lactate
[Pyr]_brain_: Pyruvate
[Glu]_brain_: Glucose
[Gly]_brain_: Glycerol
TTC: 2,3,5-triphenyltetrazolium chloride
LPR: Lactate/pyruvate ratio
DAPI: 4,6-diamino-2-phenylindole
