# Correction: Malignant infarction of the middle cerebral artery in a porcine model. A pilot study

**DOI:** 10.1371/journal.pone.0176237

**Published:** 2017-04-17

**Authors:** The PLOS ONE Staff

In Table 2, several of the columns are incorrectly labeled. The footnote for Table 2 incorrectly appears in the Results section. The journal apologizes for the error. Please see the complete, corrected Table 2 here.

**Table 2:** Metabolite values and PtiO_2_ measurements in the entire animal group during basal and ischemic periods in CORE and PENUMBRA.

Condition	TIME	PtiO_2_ (mmHg)	MICRODIALYSIS CORE (n = 5)	MICRODIALYSIS CORE (n = 3)	MICRODIALYSIS CORE (n = 3)	MICRODIALYSIS CORE(n = 3)	MICRODIALYSIS CORE (n = 3)	MICRODIALYSIS PENUMBRA (n = 3)	MICRODIALYSIS PENUMBRA (n = 3)	MICRODIALYSIS PENUMBRA (n = 3)	MICRODIALYSIS PENUMBRA (n = 3)	MICRODIALYSIS PENUMBRA (n = 3)
BASAL OR ISCHEMIA	(h)	CORE (n = 4)	Glucose (mM)	Lactate (mM)	Pyruvate (mM)	LPR	Glycerol (μM)	Glucose (mM)	Lactate (mM)	Pyruvate (mM)	LPR	Glycerol (μM)
BASAL	0	27.5 (24.4–39.2)	1.59 (0.92–2.25)	1.8 (1.2–2.5)	0.101 (0.072–0.131)	17.4 (16.1–18.8)	44.2 (23.0–65.5)	2.72	2.40	0.243	9.88	15.1
ISCHEMIA	1	0.45 (0.0–3.2)	0.70 (0.14–2.08)	8.18 (1.56–13.4)	0.074 (0.016–0.142)	64.9 (19.5–853)	77.6 (37.5–217)	1.22 (0.55–2.19)	4.54 (4.40–7.32)	0.245 (0.019–0.321)	29.9 (13.72–244.5)	148 (119–244)
ISCHEMIA	2	2.8 (1.0–4.6)	0.09 (0.00–1.88)	8.25 (7.25–9.85)	0.015 (0.000–0.050)	3241 (27.2-N/A)	319 (116–544)	1.40 (1.13–1.84)	4.06 (2.62–4.97)	0.179 (0.096–0.379)	27.2 (10.7–27.7)	103 (98.5–507)
ISCHEMIA	3	2.2 (1.0–3.1)	0.18 (0.00–2.59)	7.29 (6.85–9.43)	0.011 (0.000–0.047)	3225 (187-N/A)	462 (187–734)	1.21 (1.01–1.64)	2.94 (2.58–3.40)	0.261 (0.095–0.292)	11.7 (9.89–31.2)	111 (90.2–660)
ISCHEMIA	4	0.0 (0.0–4.7)	0.15 (0.00–2.46)	6.68 (5.84–10.7)	0.008 (0.000–0.034)	6282 (315-N/A)	545 (306–794)	1.13 (0.29–1.15)	3.06 (1.91–4.85)	0.212 (0.062–0.251)	22.9 (7.6–49.5)	115 (75.8–882)
ISCHEMIA	5	2.0 (0.0–3.4)	0.03 (0.00–2.02)	8.87 (5.16–11.2)	0.003 (0.000–0.032)	4202.0 (346-N/A)	588 (311–790)	1.13 (0.05–1.24)	2.62 (1.98–8.13)	0.251 (0.023–0.298)	27.3 (7.87–116)	141 (73.3–751)

Ischemia values are expressed as median (minimum-maximum). PtiO_2_ data of animal #5 was excluded from statistical analysis after confirming in the post-mortem study that the PtiO_2_ probe had been placed outside the area of infarction. For the sake of clarity, values under detection were considered to be 0, although the lower detection limits were 0.1 mmol/L for glucose and 0.01mmol/L for pyruvate. When pyruvate was under this limit, an L/P index was not calculated (N/A). Basal levels in the penumbra area were only available in the animal #5, and therefore minimum and maximum values in the penumbra area were not available in the basal determination.

## References

[1] Arikan F, Martínez-Valverde T, Sánchez-Guerrero Á, Campos M, Esteves M, Gandara D, et al (2017) Malignant infarction of the middle cerebral artery in a porcine model. A pilot study. PLoS ONE 12(2): e0172637 doi: 10.1371/journal.pone.0172637 28235044 10.1371/journal.pone.0172637 PMC5325275

